# Eye acupuncture therapy for insomnia: a systematic review and network meta-analysis

**DOI:** 10.3389/fneur.2025.1720073

**Published:** 2025-12-05

**Authors:** Zhenyu Shi, Shuxuan Wang, Di Sun, Hao Liu, Yaqun Wang, Cheng Xu

**Affiliations:** 1 Hangzhou First People's Hospital Xiasha Campus, Hangzhou, Zhejiang, China; 2 Tongde Hospital of Zhejiang Province Affiliated to Zhejiang Chinese Medical University (College of Integrated Traditional Chinese and Western Medicine Clinical Medicine), Hangzhou, Zhejiang, China; 3 Tongde Hospital of Zhejiang Province, Hangzhou, Zhejiang, China

**Keywords:** insomnia, eye acupuncture, systematic review, network meta-analysis, complementary and alternative medicine

## Abstract

**Objective:**

This study aims to comprehensively evaluate the efficacy and safety of eye acupuncture as a treatment modality for insomnia using advanced network Meta-analysis techniques.

**Methods:**

A systematic and thorough search was performed across eight major databases, spanning from their inception to September 01, 2025, to identify pertinent studies. In accordance with a rigorous screening process, which involved assessing titles, abstracts, and full-text articles, we performed data extraction and quality appraisal.

**Results:**

A total of 31 studies, which encompassed a diverse range of interventions, were included for detailed analysis and synthesis. The findings revealed that both eye acupuncture (EA) alone and in conjunction with other treatments, such as pharmacotherapy and non-pharmacotherapy interventions, exhibited superior efficacy compared to non-utilization of EA in ameliorating patient conditions, as evaluated through Clinical Effectiveness, Pittsburgh Sleep Quality Index score (PSQI), and Chinese Medicine Symptom Complex Score (CMSCS). The Network Meta-analysis findings revealed notable disparities in Clinical Effectiveness, PSQI, and CMSCS between interventions incorporating EA or its combination with other modalities and those not utilizing EA. Particularly, the combination of EA with body acupuncture (BA) ranked highest in reducing PSQI and CMSCS, while the combination with wrist-ankle acupuncture (WA) showed superior Clinical Effectiveness. Although the funnel plots displayed a mostly symmetrical distribution, indicating potential small sample effects and publication bias, it's crucial to interpret these results alongside clinical syndrome differentiation and treatment strategies.

**Conclusions:**

Compared to non-utilization of EA, its application demonstrates superior therapeutic efficacy in ameliorating insomnia symptoms among affected individuals. However, further validation through large-scale, multi-center, and high-quality studies is imperative to strengthen this conclusion.

**Systematic review registration:**

https://www.crd.york.ac.uk/PROSPERO/view/CRD42023491548, identifer: CRD42023491548.

## 1 Introduction

Insomnia, according to the Diagnostic and Statistical Manual of Mental Disorders, 5th edition (DSM-5), is a sleep problem defined by persistent difficulties in initiating or maintaining sleep, or experiencing early morning awakenings, despite ample opportunities for sleep, resulting in impaired daytime functioning (1). This prevalent sleep disorder affects a significant portion of the global population, with studies indicating its prevalence ranging from 10% to 30%, and in some cases, even reaching 50%−60% (2–5). For example, recent epidemiological research conducted in South Korea revealed that approximately 20% of adults experience symptoms of insomnia (6). Beyond mere disturbances in sleep, insomnia has far-reaching consequences. It can profoundly impact productivity, impair performance at work or school, elevate the risk of workplace accidents or traffic incidents, and contribute to various medical conditions, including psychiatric disorders, cognitive decline, cardiovascular ailments, and metabolic disorders (7–11). Consequently, insomnia not only compromises individuals' overall quality of life but also imposes a substantial economic burden (12).

Eye acupuncture (EA), an esteemed acupuncture modality with a rich clinical history, dates back to the early 1970s when it was pioneered by Dr. Jing-Shan Peng, a distinguished professor at Liaoning University of Traditional Chinese Medicine. Rooted in traditional Chinese medicine principles, EA operates on the premise that essence and energy flow through the 12 meridians, converging at the eyes to establish a vital connection between the meridians and the zang-fu organs. Through precise needling techniques applied to specific eye regions, the flow of qi along the meridians is stimulated, thereby regulating the functions of the zang-fu organs. This foundational concept forms the basis of EA therapy, as elucidated by Dr. Peng's comprehensive theory (13). Dr. Peng's theory anatomically divides the eye into 4 regions, comprising 8 distinct areas and 13 acupoints, assisting in both diagnostic analysis and therapeutic interventions. By balancing the functions of internal organs, clearing the meridians, and promoting the unimpeded circulation of Qi and blood, EA demonstrates significant therapeutic potential. Through the targeted stimulation of specific acupoints, EA is thought to enhance the functions of connected organs, ultimately contributing to the restoration of balance and the promotion of peaceful sleep (14).

Current hypotheses suggest that eye acupuncture may function through complex neurobiological pathways. Stimulation of the periocular region—which contains abundant trigeminal nerve endings and a dense vascular network (15)—may modulate cerebral blood flow via neurovascular coupling (16, 17), regulate sleep-wake centers such as the limbic system and hypothalamus, normalize hypothalamic-pituitary-adrenal (HPA) function to alleviate insomnia-related stress responses (18), and influence neurotransmitter systems including GABAergic to promote sleep. However, the precise mechanisms remain unclear, and comparative studies on the synergistic effects between eye acupuncture and other acupuncture techniques such as body acupuncture and wrist-ankle acupuncture are still lacking. It is worth noting that early studies (19), including a systematic review and meta-analysis conducted by our research team (20), have preliminarily explored the clinical efficacy and safety of eye acupuncture for insomnia, highlighting its advantages over conventional pharmacotherapy in terms of clinical effectiveness and reduced adverse effects. However, that earlier meta-analysis was limited to pairwise comparisons and did not evaluate the relative efficacy of different eye acupuncture combination therapies. A significant gap remains in the current literature regarding direct and indirect comparisons of the therapeutic effects of various eye acupuncture interventions. Addressing this gap, the present study employs Network Meta-Analysis to compare the efficacy and safety of multiple eye acupuncture treatments, whether administered independently or in combination with other modalities, for managing insomnia. Furthermore, it aims to rank these interventions, thereby providing valuable evidence for the development of optimized clinical treatment protocols for insomnia.

## 2 Methods

### 2.1 Protocol registration

The evaluation protocol has been published on the international prospective system evaluation registry website, PROSPERO (Registration number: CRD42023491548, URL: https://www.crd.york.ac.uk/PROSPERO/view/CRD42023491548).

### 2.2 Ethics

Since this study does not entail the recruitment of patients or the collection of personal information, ethical clearance is not required.

### 2.3 Inclusion criteria

Literature search and testing procedures were performed separately by 2 reviewers to determine eligibility. Discrepancies were discussed and settled through consensus. Initially, titles and abstracts were screened to recognize pertinent studies. Ultimately, an in-depth examination of full texts was conducted to determine whether each study satisfied the adhering to criteria:

(1) Open-label clinical trials, regardless of blinding, magazine journal, or geographical area, were consisted of. Nevertheless, studies published in Chinese or English language were just taken into consideration.
(2) Patients diagnosed with insomnia, regardless of their citizenship, race, age, sex, or period of illness, were eligible for addition.
(3) The control group encompassed individuals receiving pharmacotherapy, alternative non-pharmacotherapy interventions, or belonging to an non-intervention group (comprising placebo, waitlist, or blank control), whereas the intervention group received EA treatments either as standalone therapy or in conjunction with other modalities such as pharmacotherapy and non-pharmacotherapy therapies.
(4) In the case of multiple time points being reported in a specific study or several publications pertaining to the same research, only the record with the longest follow-up duration was considered. Additionally, if there were overlapping populations included in different reports, studies with superior quality or larger sample sizes were selected for inclusion.
(5) Simultaneously, to ensure the robustness of the study's findings, each intervention included in the analysis must comprise a minimum of two eligible studies.
(6) Studies were included if they were explicitly described as randomized controlled trials (RCTs), regardless of whether blinding was employed.
(7) Studies were required to investigate short-term interventions, defined as a total treatment duration of 4 weeks or less.

### 2.4 Exclusion criteria

(1) Non-standard assessment indicators of therapeutic effects;
(2) Poorly designed trials with vague data or insufficient original literature;
(3) Literature consisting of reviews, case records, expert opinions, systematic reviews, animal experiments, duplicate publications, or those without accessible full text.

### 2.5 Outcome indicators

Primary outcome indicators: (1) Clinical Effectiveness: this is a dichotomous outcome defined based on the percentage reduction in the Pittsburgh Sleep Quality Index (PSQI) score, which categorizes patients into effective or ineffective. Specifically, studies typically classify patients as effective when the PSQI score reduction rate reaches a predefined threshold, and ineffective when the reduction rate fails to meet this threshold. The specific thresholds and calculation methods for “effective” and “cure” varied across included studies. Detailed information on the effectiveness definitions of each study is summarized in Supplementary Table 1. (2) PSQI, which is a self-rated questionnaire assessing sleep quality over 1 month. Its seven components yield a global score ranging from 0 to 21, where higher scores indicate poorer sleep quality. A global score >5 distinguishes “poor” from “good” sleepers, and a score reduction post-intervention signifies improvement. Secondary outcome indicators: Chinese medicine symptom complex score (CMSCS).

### 2.6 Search strategy

The study employed a comprehensive search strategy, incorporating both subject-specific terminology and free-text wording, across a series of English databases including of PubMed, Embase, and the Cochrane Library. In addition, the search encompassed other databases such as China National Knowledge Infrastructure (CNKI), Wanfang, China Science and Technology Journal Database, and China Biology Medicine (CBM) to ensure coverage and retrieval of relevant literature. The search period extended up to September 01, 2025. A mix of subject terms and free-text terms was used for the search method. Chinese search terms comprised a range of descriptors related to insomnia, such as Insomnia, Sleeplessness, Insomnia Disorder, Sleep Initiation and Maintenance Disorders, alongside terms for eye acupuncture. English search terms encompassed various iterations of insomnia and diverse forms of eye acupuncture. Table 1 illustrates the search strategy employed for PubMed.

**Table 1:** Search strategy of PubMed.

**Number**	**Search terms**
#1	“Sleep Initiation and Maintenance Disorders”[Mesh]
#2	((((((((((Insomnia[Title/Abstract]) OR (Sleeplessness[Title/Abstract])) OR (Insomnias[Title/Abstract])) OR (Insomnia Disorder[Title/Abstract])) )) OR (Sleep Initiation Dysfunction[Title/Abstract])) OR (Dysfunctions, Sleep Initiation[Title/Abstract])) OR (Sleep Initiation Dysfunctions[Title/Abstract])) OR (Disorders of Initiating[Title/Abstract] AND Maintaining Sleep[Title/Abstract])) OR (Sleep Initiation[Title/Abstract] AND Maintenance Disorders[Title/Abstract])
#3	#1 OR #2
#4	“Eye Acupuncture”[Mesh] - Schema: all
#5	(((Eye Acupuncture[Title/Abstract]) OR (Eye Acupuncture therapy[Title/Abstract])) OR (Eye Acupuncture points[Title/Abstract]))
#6	#4 OR #5
#7	“Randomized Controlled Trials as Topic”[Mesh]
#8	(((Controlled clinical trial[Title/Abstract]) OR (Randomized[Title/Abstract])) OR (Randomly[Title/Abstract])) OR (Trial[Title/Abstract])
#9	#7 OR #8
#10	#3 AND #6 AND #9

### 2.7 Data screening and extraction

The researchers complied with the Cochrane Collaboration Systematic Review Manual Version 5.0. Initially, two researchers utilized EndNote X20 bibliographic management software to evaluate the literature based on the PRISMA flowchart. They excluded duplicates and clearly unqualified literature according to the predefined criteria and then reviewed the full text of the remaining articles. During the second screening stage, two researchers independently assessed the articles to determine if they met the inclusion criteria. Any inconsistencies were resolved through consultation with a third researcher, particularly for studies that posed challenges in decision-making. Important information including author names, publication year, country of origin, sample size, patient demographics (age, disease duration), along with details pertaining to control and intervention protocols, outcome variables, and treatment duration, were extracted using Excel 2013 software. For studies that did not report complete data, we attempted to acquire missing information by email or other means of communication with the authors.

### 2.8 Quality of literatures

The risk of bias for the included studies was assessed using the Cochrane Collaboration's tool. Two researchers independently evaluated the following domains for each study: (1) random sequence generation; (2) allocation concealment; (3) blinding of participants and personnel; (4) blinding of outcome assessment; (5) incomplete outcome data; (6) selective reporting; (7) other bias. Judgments were categorized as “low risk,” “some concerns,” or “high risk” for each domain. The reviewers then cross-checked their assessments. Any discrepancies were resolved through discussion, or by consultation with a third researcher when consensus could not be reached initially.

### 2.9 Statistical analysis

The frequentist method using random effect models was employed to conduct network meta-analyses (NMAs) of randomized controlled trials. Statistical analyses were conducted using the network package based on Stata MP16.0. Continuous variables (PSQI, CMSCS) were expressed as standardized mean differences (SMD) with their 95% confidence intervals (CI), while dichotomous variables (Clinical Effectiveness) were expressed as risk ratios (RR) with their 95% CI. For studies involving three or more arms, the study will be split and error-corrected before inclusion in the network meta-analysis. Since control groups are prone to repeated use, their sample sizes will be proportionally reduced based on the number of splits and comparisons. To evaluate transitivity, we examined the distributions of key study characteristics across comparison groups; any detected differences were explored via meta-regression to assess their impact on the results. We assessed statistical inconsistency between direct and indirect evidence using both global and local tests. As a global approach, we used a design-by-treatment interaction model to investigate the inconsistency in the entire network. We evaluated local inconsistency based on the node-splitting method. We produced league tables with 95% confidence intervals and calculated the surface under the review the cumulative ranking curve (SUCRA) for each intervention. Publication bias was assessed using comparison-adjusted funnel plots. Regarding missing or incomplete data, studies with essential missing outcome data that could not be retrieved through author contact or calculated from available statistics were excluded during the full-text screening phase to ensure the integrity of the analysis.

## 3 Results

### 3.1 Study selection

**Figure 1:**
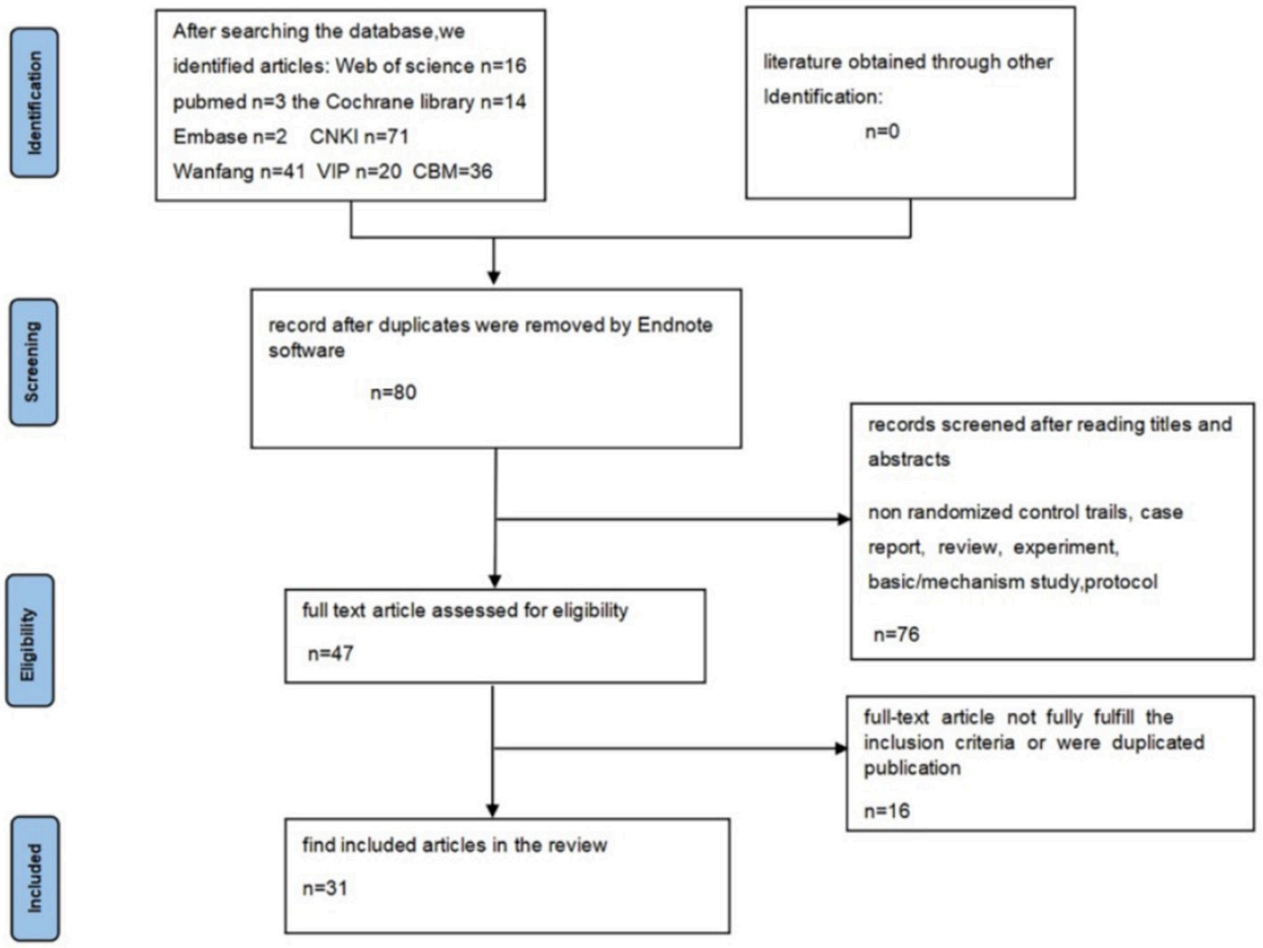
Flowchart of study screening process.

Figure 1 presents a flowchart of the literature search and testing procedure, showcasing the initial search results and the subsequent selection of studies for inclusion in the review. Initially, a search generated 203 relevant publications, from which 80 duplicate articles were identified and ultimately removed after importation into Endnote. Upon assessing titles and abstracts, an additional 76 articles were omitted. A detailed assessment of the full-text articles led to the exclusion of 16 studies due to inconsistencies in treatment protocols, resulting in a final sample of 31 (21–51) studies.

### 3.2 Study characteristics

In the selected studies, a total of 9 interventions were examined. The combined sample size amounted to 2,293 patients, distributed as 1,165 in the experimental groups and 1,128 in the control groups. The control group comprised 4 interventions, namely western medicine (WM), traditional Chinese medicine (TCM), body acupuncture (BA), and traditional Chinese medicine combined with western medicine (TCM+WM). Intervention measures in the experimental group included the use of EA alone, eye acupuncture combined with body acupuncture (EA+BA), eye acupuncture combined with traditional Chinese medicine (EA+TCM), eye acupuncture combined with western medicine (EA+WM), and eye acupuncture combined with wrist-ankle acupuncture (EA+WA), totaling 5 types. Detailed characteristics of the included studies can be found in Table 2.

**Table 2:** Basic characteristics.

**Study ID**	**Age, yr**		**Sample size**		**During of disease**		**Intervention**		**Intervention period**	**Outcome measure**
**N**	**T**	**C**	**T**	**C**	**T**	**C**	**T**	**C**	**T/C**	**T/C**
Zhao (2022)	52.17 ± 12.76 (19–64)	49.07 ± 10.01 (27–64)	30	30	6.37 ± 2.414 mo (1–11)	5.37 ± 2.385 mo (1-10)	EA+WM	WM	4wk	PSQI, CMSCS, CE
Li (2022)	56.29 ± 7.64	55.93 ± 7.52	42	42	16.47 ± 4.26 mo	15.89 ± 4.37 mo	EA+BA	BA	4wk	PSQI, CMSCS
Zhang (2021)	55.38 ± 0.43 (47-84)	56.75 ± 0.32 (44–83)	40	40			EA	BA	2wk	PSQI
Bai and Hai (2021)	50.24 ± 6.14 (36–63)	50.52 ± 5.91 (33–64)	40	40	1.50 ± 0.30 yr (0.5–3)	1.48 ± 0.10 yr (0.5–3)	EA+TCM	TCM	3wk	PSQI, CE
Bai (2021)	42.20 ± 7.48 (32–55)	43.57 ± 6.77 (33–57)	30	30	12.47 ± 4.79 wk (5–22)	12.70 ± 4.49 wk (4–21)	EA+TCM	TCM	21 d	PSQI, CMSCS, CE
Luo (2020)	60.23 ± 2.16	61.67 ± 2. 73	30	30	40.37 ± 2. 50 d	41.12 ± 1. 32 d	EA+BA	BA	10 d	PSQI, CE
Qin (2019)	37.2 ± 4.8 (20–70)	36.8 ± 4.5 (22–68)	45	45	1.5 ± 0.7 yr (2 mo−3 yr)	1.6 ± 0.6 yr (3 mo−3 yr)	EA+WM	WM	4wk	PSQI, CE
Wu (2019)	38.17 ± 11.57 (19–56)	38.83 ± 11.114 (20–55)	30	30	8.43 ± 3.892 mo (3–16)	8.90 ± 3.818 mo (4–17)	EA+BA	BA	15 d	PSQI, CMSCS
Li Qin (2019)	47.17 ± 12.22	48.17 ± 11.66	30	30	7.63 ± 1.88 mo	7.70 ± 1.51 mo	EA+TCM	WM	4wk	PSQI, CMSCS
Li and Wang (2019)	51.20 ± 16.8 (22–78)	50.10 ± 13. 62 (21–75)	42	38	6.56 ± 5. 15 mo (0. 57–20)	7.70 ± 3. 56 mo (0.67–15)	EA	BA	14 d	PSQI, CE
Li (2019)	50.27 ± 9.51 (30–65)	51.10 ± 10.82 (32–65)	30	30	14.57 ± 7.80 mo (2–36)	14.37 ± 8.95 mo (1–40)	EA	BA	10 d	PSQI, CE
Huang (2019)	41–70	43–72	40	40	11 mo−5 yr	8 mo−5 yr	EA+TCM	TCM	10 d	PSQI, CE
Zhang (2018)	49.3 ± 7.65	51.1 ± 7.12	30	30	12.56 ± 8.72 wk	11.87 ± 9.15 wk	EA+WA	BA	20 d	CE
Cao (2020)	49.56 ± 6.32 (22–71)	50.06 ± 6.71 (20–73)	50	50	10.23 ± 2.58wk (3–20)	9.85 ± 2.18 wk (3–19)	EA+WA	BA	20 d	CE
Xie (2018)	49.5 ± 6.6 (22–78)	49.6 ± 6.5 (23–76)	30	30	2.1 ± 0.3 yr (1 mo−3 yr)	2.2 ± 0.4 yr (2 mo−3 yr)	EA+BA	BA	20 d	PSQI
Wang and Wang (2018)	50.50 ± 17.80 (20–76)	49.10 ± 13.62 (24–74)	30	30	7.56 ± 4.15 mo (0.67–15)	7.70 ± 3.56 mo (0.67–15)	EA+TCM	TCM	28 d	PSQI, CE
Wang (2018)	54.50 ± 13.76 (23–75)	53.20 ± 11.89 (24–75)	30	30	8.67 ± 3.53 mo (2–15)	9.22 ± 4.25 mo (3–19)	EA+TCM	WM	28 d	PSQI, CMSCS, CE
Hu (2018)	62.5 ± 7.3 (46–80)		30	30	24.6 ± 7.6 d (5–67)		EA+TCM	TCM+WM	4wk	PSQI, CE
Guo (2018)	65.233 ± 11.285	63.50 ± 10.92	30	30			EA+WM	WM	10 d	PSQI
Wang (2017)	49.22 ± 12.37	47.11 ± 11.37	37	37	1.68 ± 1.94 yr	1.97 ± 2.31 yr	EA+TCM	TCM	20 d	PSQI, CMSCS
Liu (2017)	55 ± 3	54 ± 4	30	30	8.5 ± 6.2 mo	8.6 ± 6.0 mo	EA	BA	30 d	CE
Li (2017)	34.73 ± 10.62 (18–57)	34.2 ± 10.22 (19–56)	30	30	9.00 ± 2.51 mo (5–14)	9.03 ± 2.48 mo (5–13)	EA+BA	EA	12 d	PSQI, CMSCS
		35.53 ± 9.98 (18–56)		30		9.17 ± 2.88 mo (5–14)		BA		
Ma (2016)	40.10 ± 9.72 (27–63)	41.67 ± 9.82 (28–60)	30	30	11.93 ± 9.56 mo (1–30)	11.60 ± 9.31 mo (1–36)	EA	TCM	14 d	PSQI, CMSCS, CE
Tian (2015)	64.5 ± 6.9 (53–77)	61.1 ± 7.6 (49–76)	60	60	11.4 ± 6.5 mo	9.1 ± 7.8 mo	EA+BA	WM	30 d	PSQI, CE
Cheng (2015)	48.58 ± 13.47	47.22 ± 13.35	36	36	12.94 ± 8.62 mo	12.75 ± 8.18 mo	EA+BA	BA	20 d	CE
Xu (2014)	49.11 ± 3.62 (31–60)	49.5 ± 4.01 (33–60)	45	45	4.83 ± 3.79 mo (1–22)	4.40 ± 3.01 mo (1–23)	EA	TCM+WM	14 d	PSQI, CE
Zhang (2013)	45.43 ± 2.66 (18–69)	49.23 ± 2.50 (21–70)	30	30	7.53 ± 2.36 yr (1–20)	8.27 ± 2.70 yr (1–20)	EA+TCM	TCM	14 d	CE
Liu (2013)	43 ± 2.3 (20–70)	41 ± 1.8 (22–68)	40	40	4 ± 0.4 yr (2 mo−8 yr)	5 ± 0.7 yr (3 mo−10 yr)	EA	TCM	4wk	CE
Cui (2011)	45 (20–75)	42 (18–75)	40	40	15 d−18 yr	18 d−16 yr	EA+TCM	TCM	10 d	CE
Luo (2010)	41.50 ± 8.85 (25–64)	41.45 ± 8.45 (35–62)	30	29	9.67 ± 5.69 wk (4–24)	9.17 ± 4.75 wk (4–22)	EA+TCM	TCM	20 d	PSQI, CMSCS, CE
Huang (2010)	18–70	22–69	68	66	0.5–10 yr	8 mo−11 yr	EA	WM	2wk	CE

BA, body acupuncture; CE, Clinical effectiveness; CMSCS, Chinese medicine symptom complex score; C, control group; d, day; EA, eye Acupuncture; WM, western medicine; WA, wrist-ankle acupuncture; mo, month; PSQI, Pittsburgh sleep quality index; TCM, Traditional Chinese Medicine; T, treatment group; wk, week; yr, year.

### 3.3 Risk of bias

**Figure 2:**
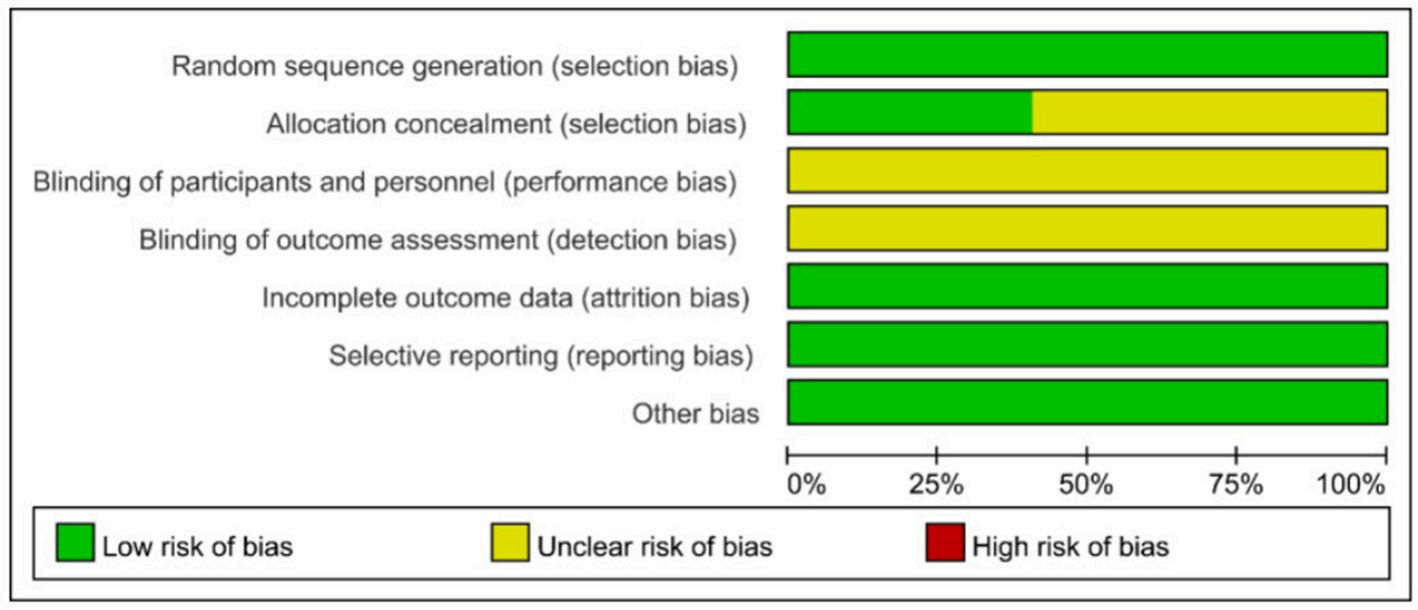
Percentage of projects at risk of bias included in study.

The methodological quality graph (Figure 2) shows that 11 trials (22, 24, 25, 30, 31, 36, 37, 39, 42, 46, 51) used random number methods and 1 study (38) used a random method generated using computer SAS software. The remaining 19 trials (21, 23, 26–29, 32–35, 40, 41, 43–45, 47–50) used “random allocation” but did not specify the method of generating randomized sequences. None of the trials mentioned whether blinding was used or reported participant dropouts, thus indicating a potential risk of performance and detection bias, as well as a high risk of attrition bias. A detailed summary of the risk of bias assessment for each individual study across all domains is presented in Table 3 to enhance transparency.

**Table 3:** Summary table of the risk of bias.

**Study ID**	**Random sequence generation**	**Allocation concealment**	**Blinding of participants and personnel**	**Blinding of outcome assessment**	**Incomplete outcome data**	**Selective reporting**	**Other bias**
Zhao (2022)	Low	Unclear	Unclear	Unclear	Low	Low	Low
Li (2022)	Low	Low	Unclear	Unclear	Low	Low	Low
Zhang (2021)	Low	Unclear	Unclear	Unclear	Low	Low	Low
Bai and Hai (2021)	Low	Low	Unclear	Unclear	Low	Low	Low
Bai (2021)	Low	Low	Unclear	Unclear	Low	Low	Low
Luo and Yang (2020)	Low	Unclear	Unclear	Unclear	Low	Low	Low
Qin and Chen (2019)	Low	Unclear	Unclear	Unclear	Low	Low	Low
Wu (2019)	Low	Unclear	Unclear	Unclear	Low	Low	Low
Li Qin (2019)	Low	Unclear	Unclear	Unclear	Low	Low	Low
Li and Wang (2019)	Low	Low	Unclear	Unclear	Low	Low	Low
Li (2019)	Low	Low	Unclear	Unclear	Low	Low	Low
Huang (2019)	Low	Unclear	Unclear	Unclear	Low	Low	Low
Zhang (2018)	Low	Unclear	Unclear	Unclear	Low	Low	Low
Cao (2020)	Low	Unclear	Unclear	Unclear	Low	Low	Low
Xie (2018)	Low	Unclear	Unclear	Unclear	Low	Low	Low
Wang and Wang (2018)	Low	Low	Unclear	Unclear	Low	Low	Low
Wang (2018)	Low	Low	Unclear	Unclear	Low	Low	Low
Hu (2018)	Low	Low	Unclear	Unclear	Low	Low	Low
Guo (2018)	Low	Unclear	Unclear	Unclear	Low	Low	Low
Wang (2017)	Low	Low	Unclear	Unclear	Low	Low	Low
Li (2017)	Low	Unclear	Unclear	Unclear	Low	Low	Low
Ma (2016)	Low	Low	Unclear	Unclear	Low	Low	Low
Tian (2015)	Low	Unclear	Unclear	Unclear	Low	Low	Low
Cheng (2015)	Low	Unclear	Unclear	Unclear	Low	Low	Low
Xu (2014)	Low	Low	Unclear	Unclear	Low	Low	Low
Cheng (2014)	Low	Unclear	Unclear	Unclear	Low	Low	Low
Zhang (2013)	Low	Low	Unclear	Unclear	Low	Low	Low
Liu (2013)	Low	Unclear	Unclear	Unclear	Low	Low	Low
Cui (2011)	Low	Unclear	Unclear	Unclear	Low	Low	Low
Luo (2010)	Low	Unclear	Unclear	Unclear	Low	Low	Low
Huang (2010)	Low	Unclear	Unclear	Unclear	Low	Low	Low

### 3.4 Meta-analysis

#### 3.4.1 Primary outcome

##### 3.4.1.1 Clinical effectiveness

**Figure 3:**
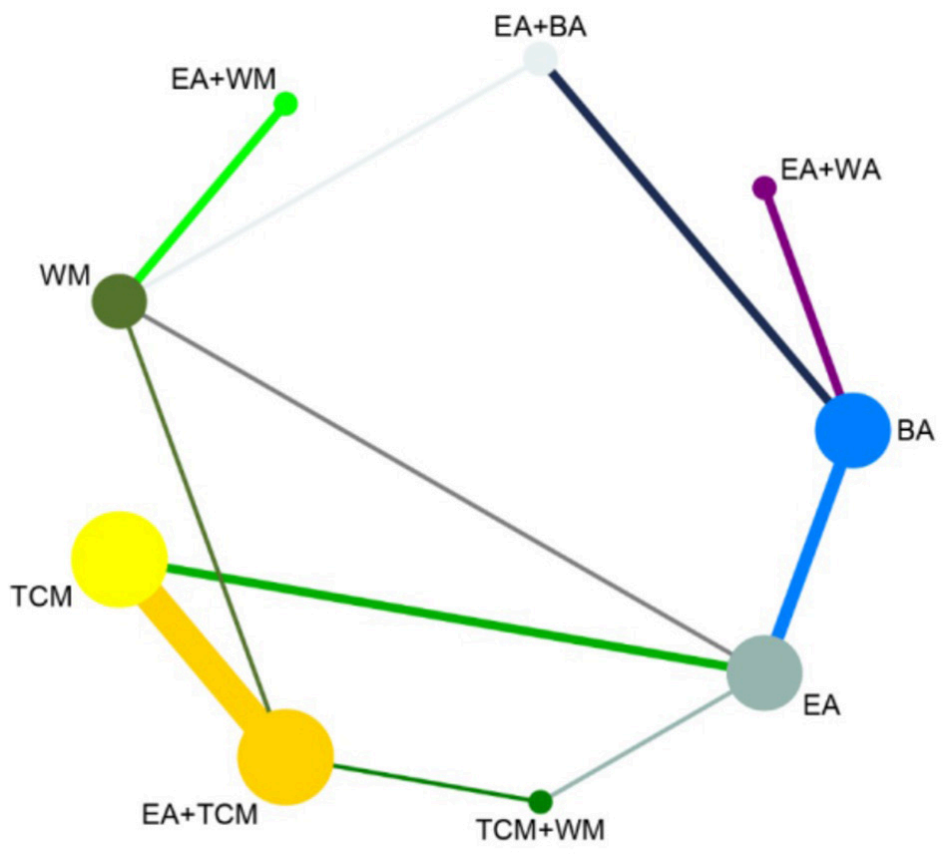
Network diagram of clinical effectiveness.

The network meta-analysis covered 9 treatments. Findings for suggested that EA+TCM had the highest number of studies (9) and the largest sample size (479 patients) compared to TCM alone. Additionally, the evidence network formed a closed loop. For a diagram of the evidence network, please refer to Figure 3.

**Figure 4:**
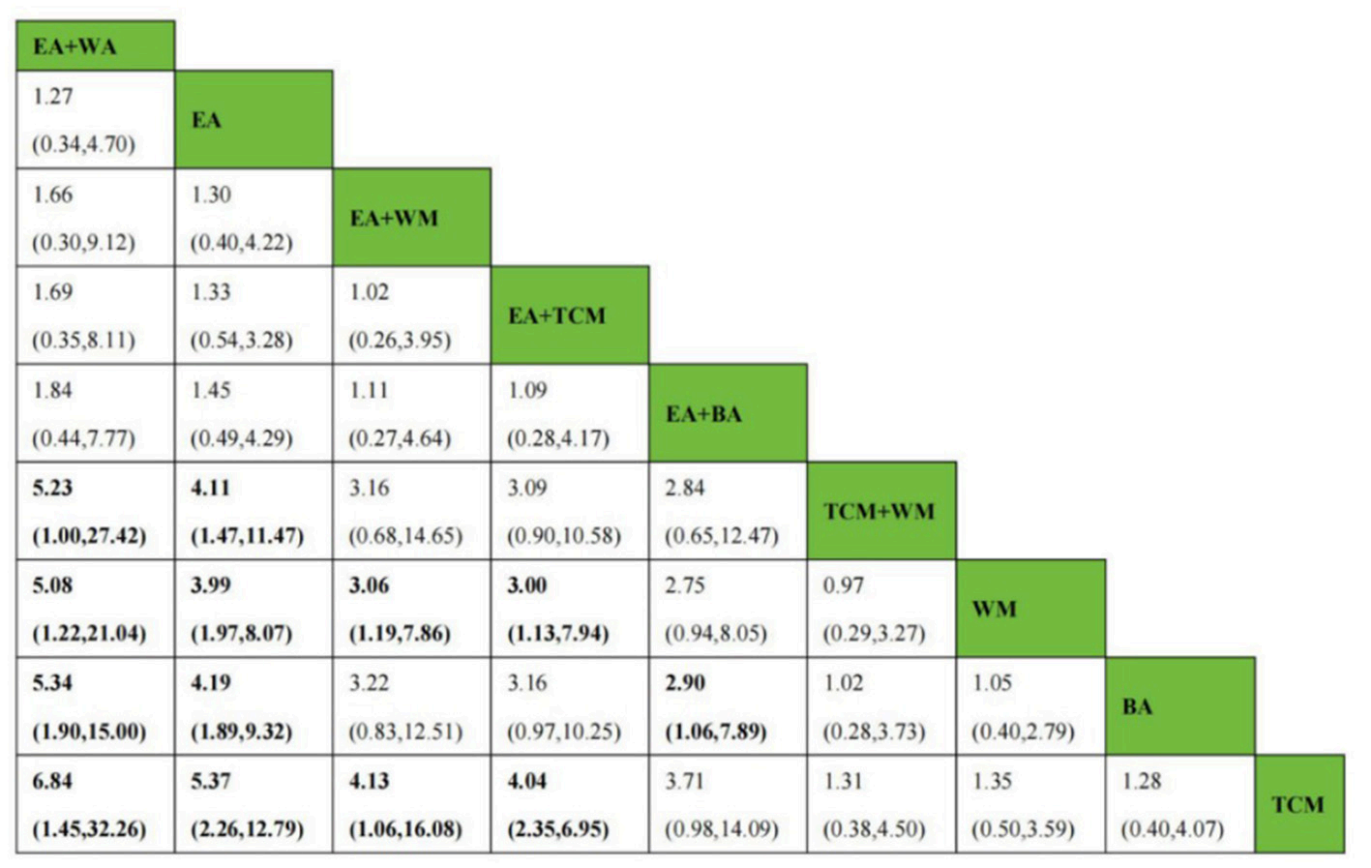
League figures of clinical effectiveness.

After the formation of a closed loop, the analysis initially used an inconsistent model to examine Clinical Effectiveness. The results revealed a *P*-value of 0.61 (*P* > 0.05), indicating non-significant inconsistency. Subsequently, the node-splitting method was employed to assess local inconsistency, revealing no significant differences between direct and indirect comparisons of treatment procedures (*P* > 0.05), suggesting acceptable consistency. The network meta-analysis was then conducted using a consistent model. Out of the 36 pairwise comparisons generated, 13 were statistically significant. Notably, all interventions utilizing EA demonstrated superior efficacy in enhancing Clinical Effectiveness compared to non-EA interventions. Specifically, EA+WA, EA, EA+WM, and EA+TCM exhibited superior efficacy to TCM alone in improving Clinical Effectiveness. Moreover, the efficacy of EA+WA, EA, EA+WM, and EA+TCM in treating insomnia was superior to that of WM alone. Additionally, EA+WA, EA, and EA+BA were more effective in treating insomnia compared to BA alone, while EA+WA and EA were superior to TCM+WM. Other comparative differences did not reach statistical significance. Please refer to Figure 4 for league tables.

**Figure 5:**
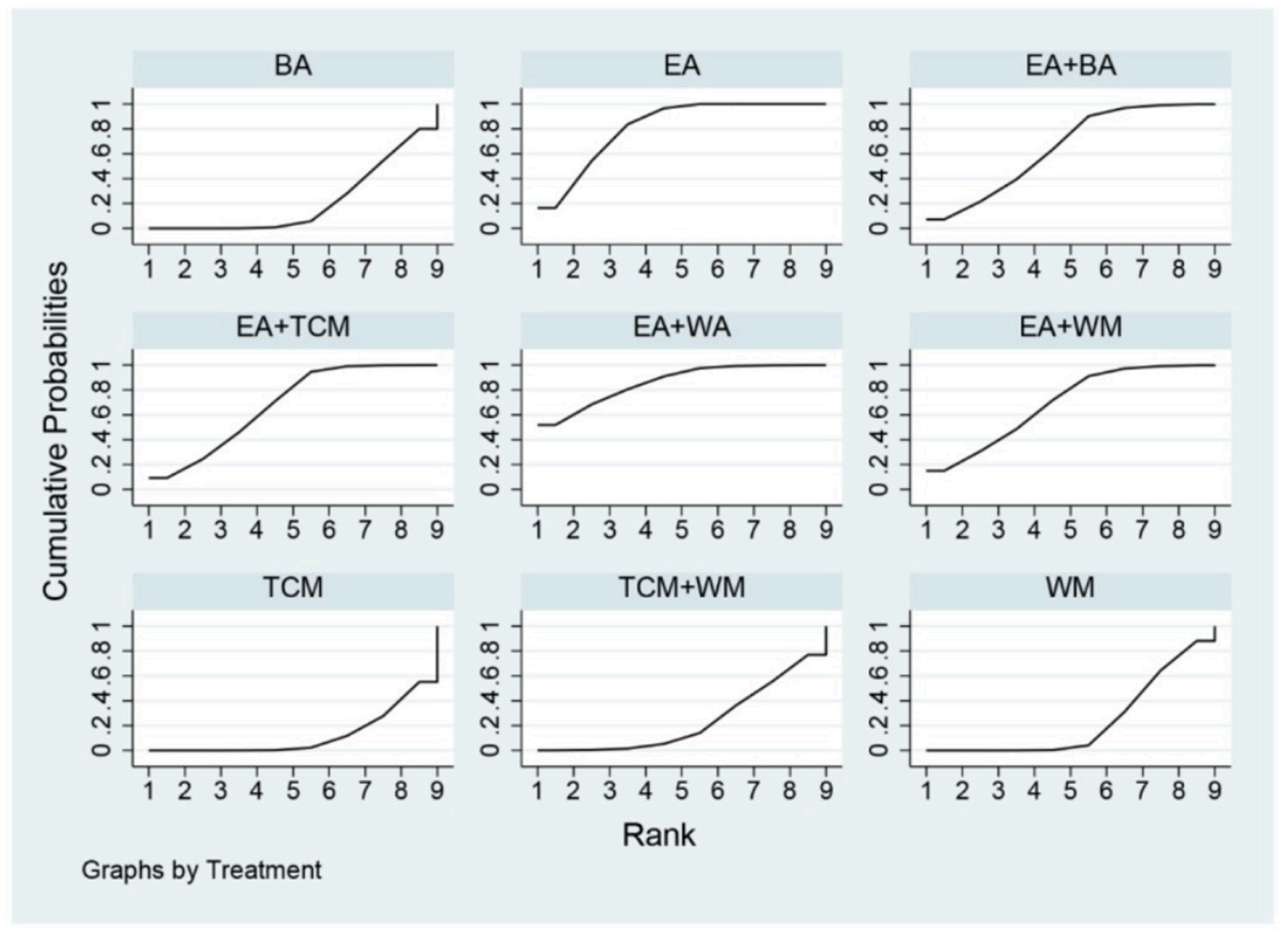
SUCRA probability ranking of clinical effectiveness.

**Figure 6:**
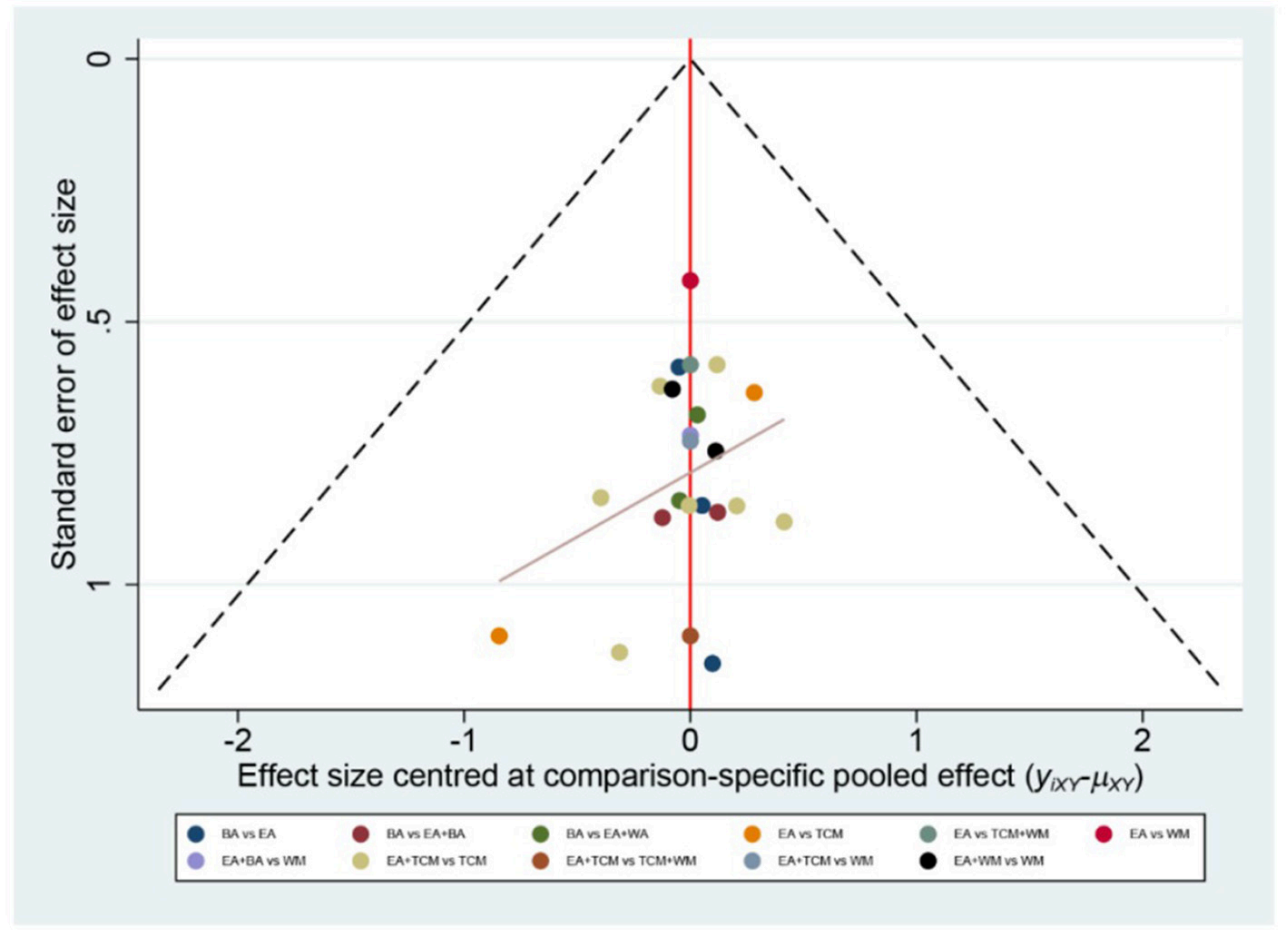
Comparative-corrected funnel plot of clinical effectiveness.

The probability ranking based on SUCRA is outlined as follows: EA+WA (86.1%) > EA (81.4%) > EA+WM (69.2%) > EA+TCM (68%) > EA+BA (64.7%) > TCM+WM (23.7%) > WM (23.5%) > BA (21.2%) > TCM (12.2%). These results suggest that eye acupuncture combined with wrist-ankle acupuncture is the most effective intervention for enhancing Clinical Effectiveness. The SUCRA probability ranking is illustrated in Figure 5. Additionally, the funnel plot in Figure 6 displayed a broadly symmetrical distribution for most comparisons, suggesting a low likelihood of significant publication bias.

##### 3.4.1.2 PSQI

**Figure 7:**
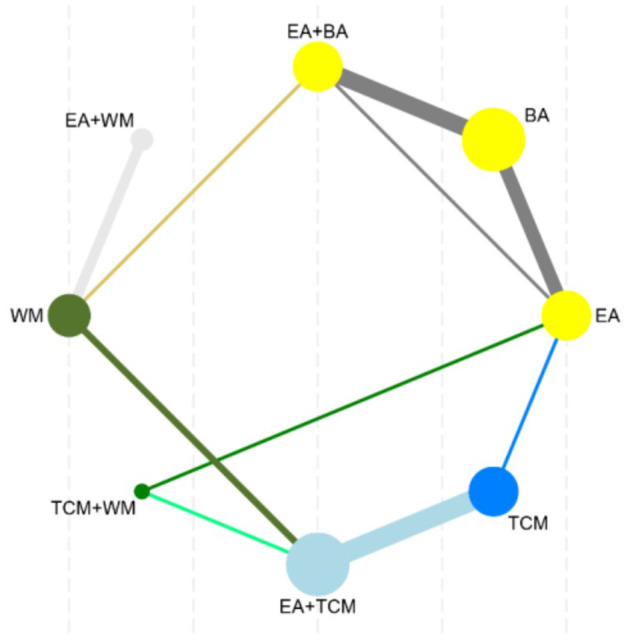
Network diagram of PSQL.

After performing a subsequent network meta-analysis involving 8 interventions, it was found that EA+TCM had the highest number of studies (7) and the largest sample size (413 patients) compared to TCM alone. Furthermore, a closed loop was formed in the evidence network, as illustrated in Figure 7.

**Figure 8:**
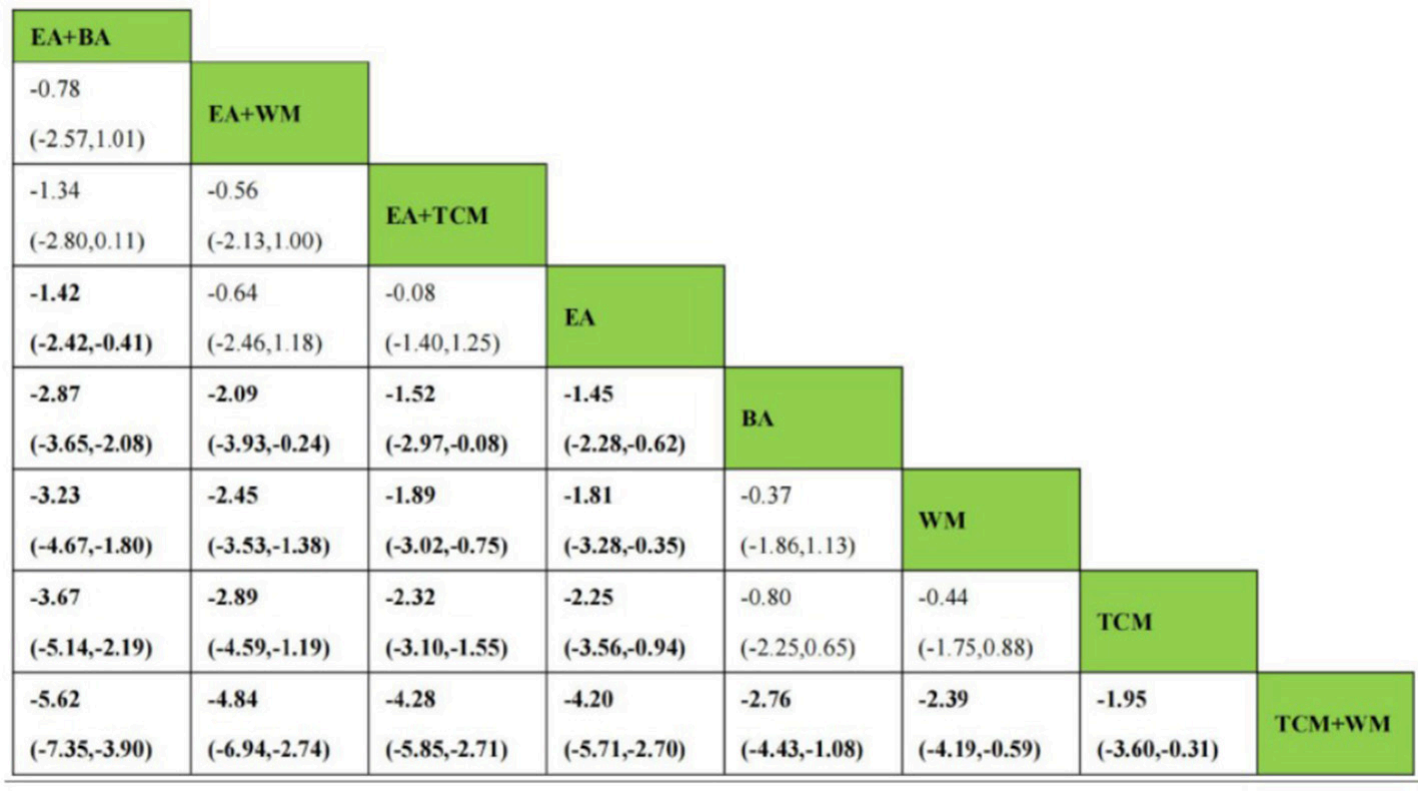
League figure of PSQI.

As a closed loop was formed, the PSQI score underwent an initial assessment using an inconsistent model. Findings disclosed a non-significant inconsistency with *P* = 0.45 (*P* > 0.05). Subsequently, the node-splitting method was used to analyze local inconsistency, showing no significant differences between direct and indirect comparisons of treatment measures (*P* > 0.05), indicating strong consistency. Following this, a consistent model was employed for network meta-analysis. Out of the 28 pairwise comparisons generated, 21 were statistically significant. Please refer to Figure 8 for league tables. Regarding the reduction of the PSQI score, interventions involving EA or EA combined with other modalities exhibited superior efficacy compared to non-EA interventions. Among them, EA+BA, EA+WM, EA+TCM, EA, BA, WM, and TCM demonstrated greater effectiveness than TCM+WM in reducing the PSQI score. Additionally, EA+BA, EA+WM, EA+TCM, EA, and BA interventions were more effective in treating insomnia compared to TCM alone. Moreover, the efficacy of EA+BA, EA+WM, EA+TCM, EA, and BA in treating insomnia was superior to that of WM alone, while the efficacy of EA+BA, EA+WM, EA+TCM, and EA was superior to BA alone. Other comparative differences did not achieve statistical significance.

**Figure 9:**
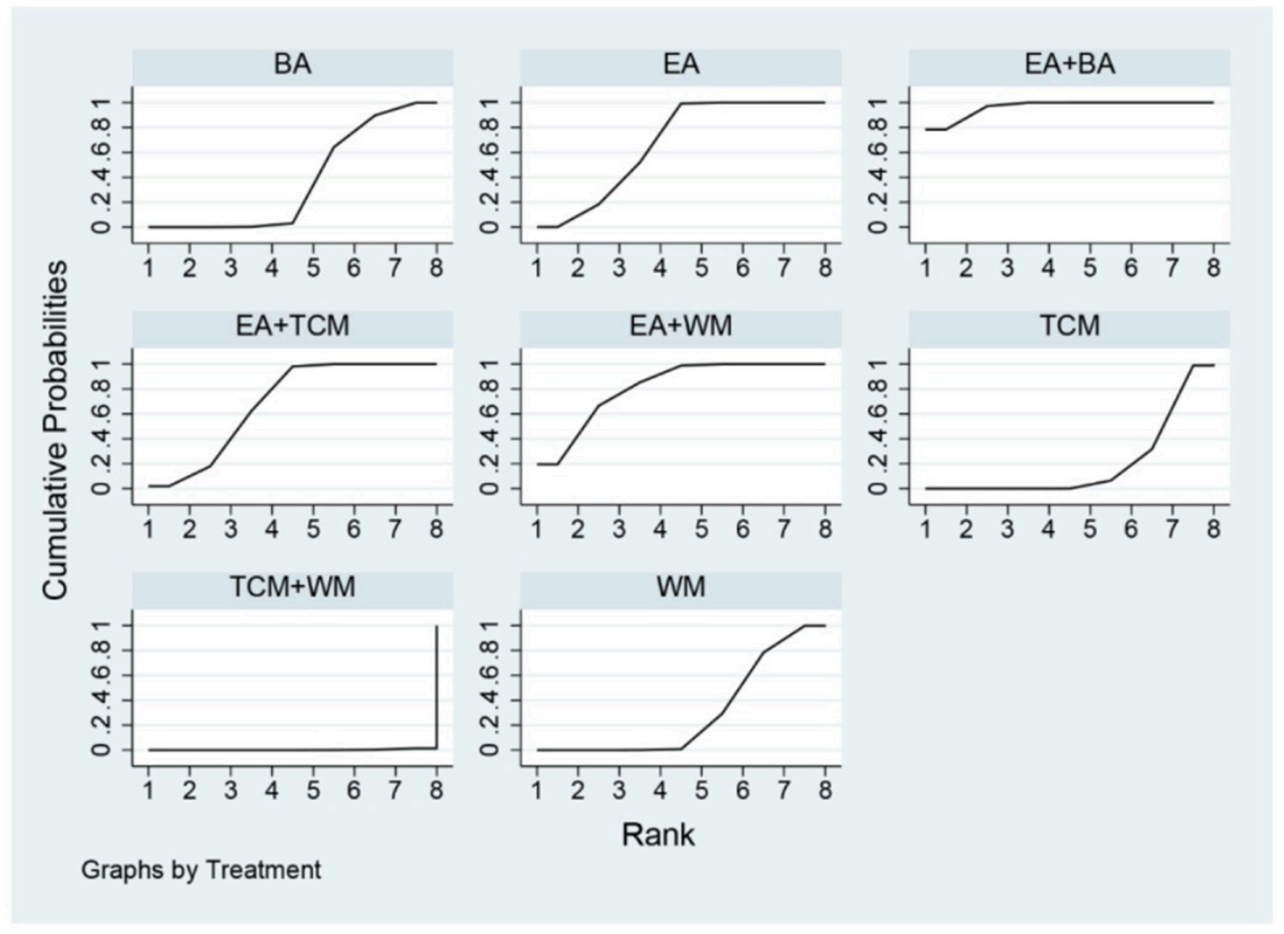
SUCRA probability ranking of PSQI.

**Figure 10:**
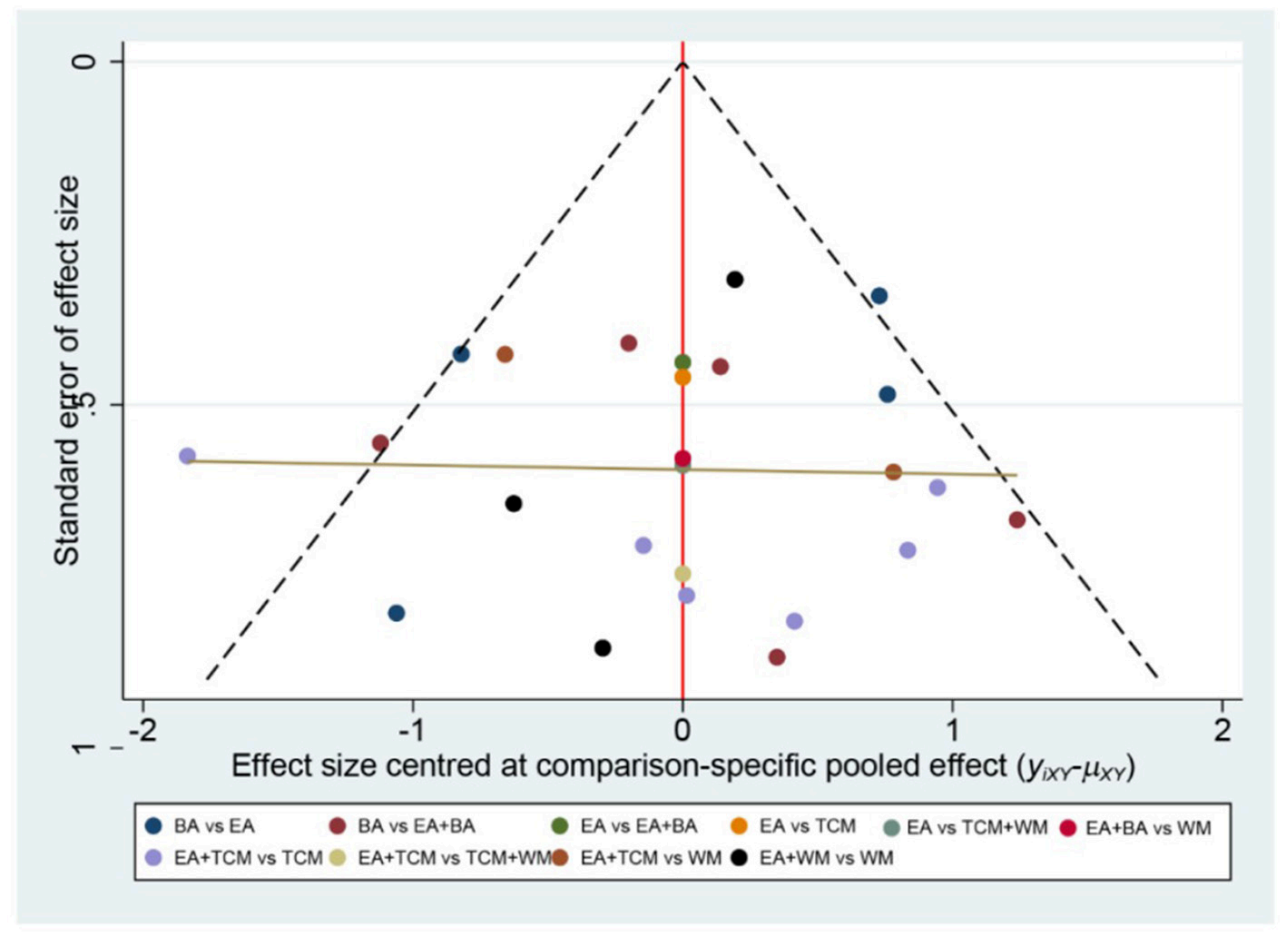
Comparative-corrected funnel plot of PSQI.

The probabilities ranked by SUCRA were as follows: EA+BA (96.5%) > EA+WM (86.4%) > EA+TCM (69.6%) > EA (67.1%) > BA (36.7%) > WM (29.7%) > TCM (19.6%) > TCM+WM (0.2%). These findings indicate that the combination of eye acupuncture with body acupuncture appears to be the most promising intervention. The SUCRA probability ranking is shown in Figure 9. Moreover, the plot in Figure 10 showed asymmetry, suggesting the potential for publication bias or small-study effects.

#### 3.4.2 Secondary outcome

##### 3.4.2.1 CMSCS

**Figure 11:**
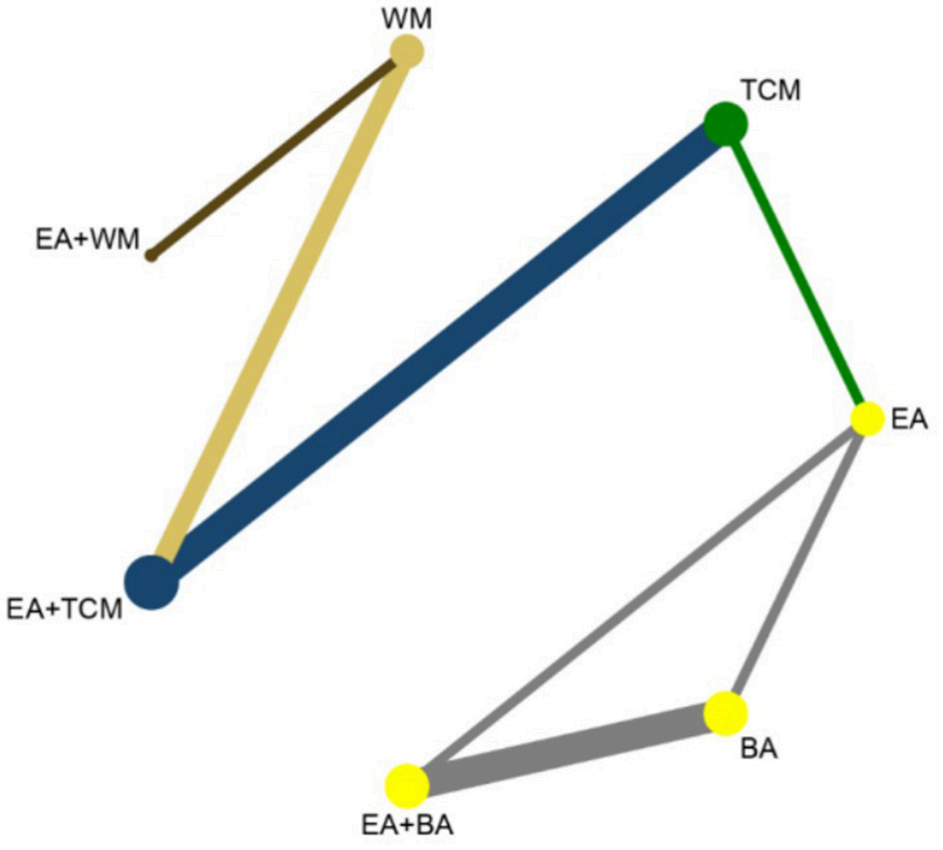
Network diagram of CMSCS.

The analysis incorporated 10 studies involving 7 interventions. Among these, EA+TCM was the most studied treatment, with 3 studies and the largest sample size of 193 patients compared to TCM alone. Furthermore, the evidence network diagram showed a closed-loop formation, as shown in Figure 11.

**Figure 12:**
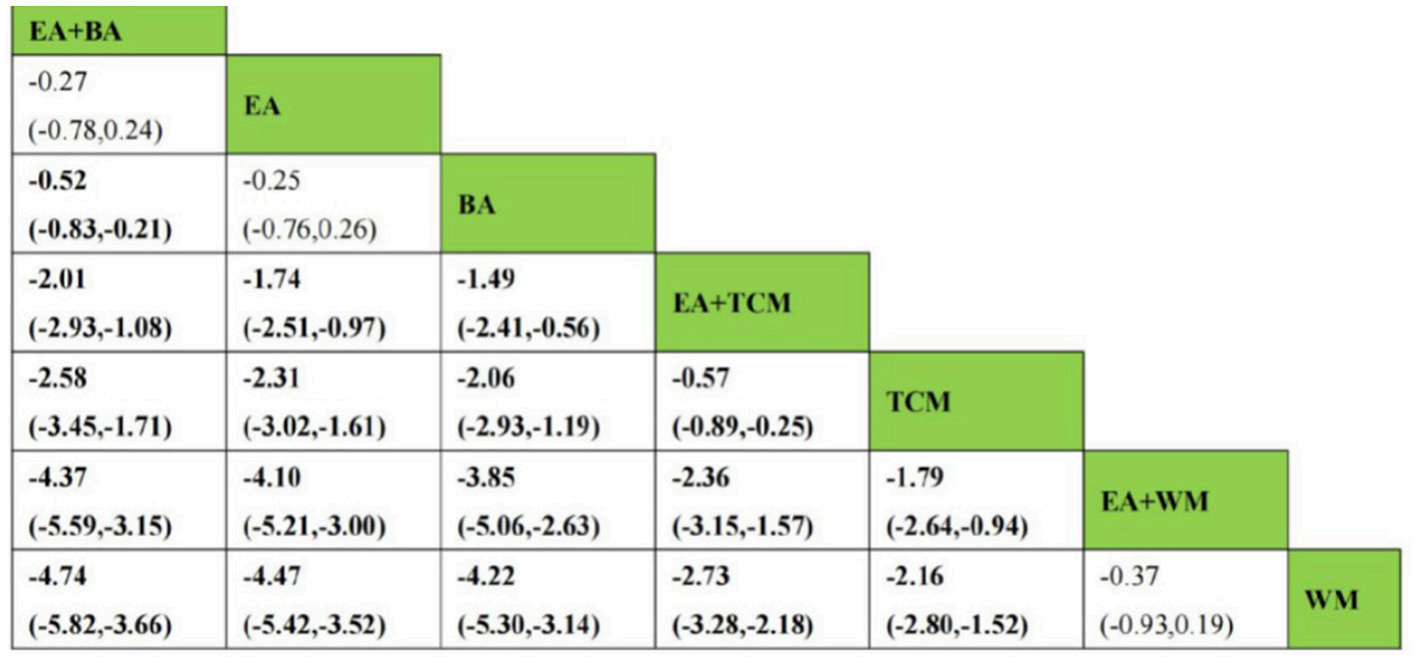
League figure of CMSCS.

Given the formation of a closed loop, the analysis of CMSCS initially used an inconsistent model. The findings revealed a *P*-value of 0.60 (*P* > 0.05), indicating no statistically significant inconsistency. Additionally, employing the node-splitting method to conduct a local inconsistency examination showed no significant differences between direct and indirect comparisons of intervention procedures (*P* > 0.05), thus confirming strong consistency. In the network meta-analysis, a total of 21 pairwise comparisons were generated, 18 of which were statistically significant. The league tables are shown in Figure 12. Notably, interventions involving EA+BA, EA, BA, EA+TCM, and TCM outperformed WM in reducing CMSCS. Furthermore, EA+BA, EA, BA, EA+TCM, and TCM were more effective in treating insomnia compared to EA+WM. Moreover, the efficacy of EA+BA, EA, and BA in treating insomnia was superior to that of EA+TCM. It is noteworthy that EA did not significantly reduce CMSCS compared to BA (*P* > 0.05). Additionally, there was no statistically significant difference found between EA+BA and EA in terms of reducing CMSCS.

**Figure 13:**
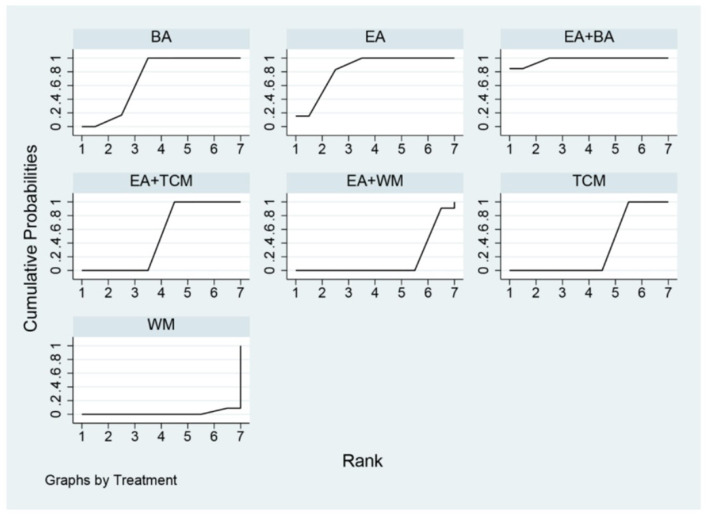
SUCRA probability ranking of CMSCS.

**Figure 14:**
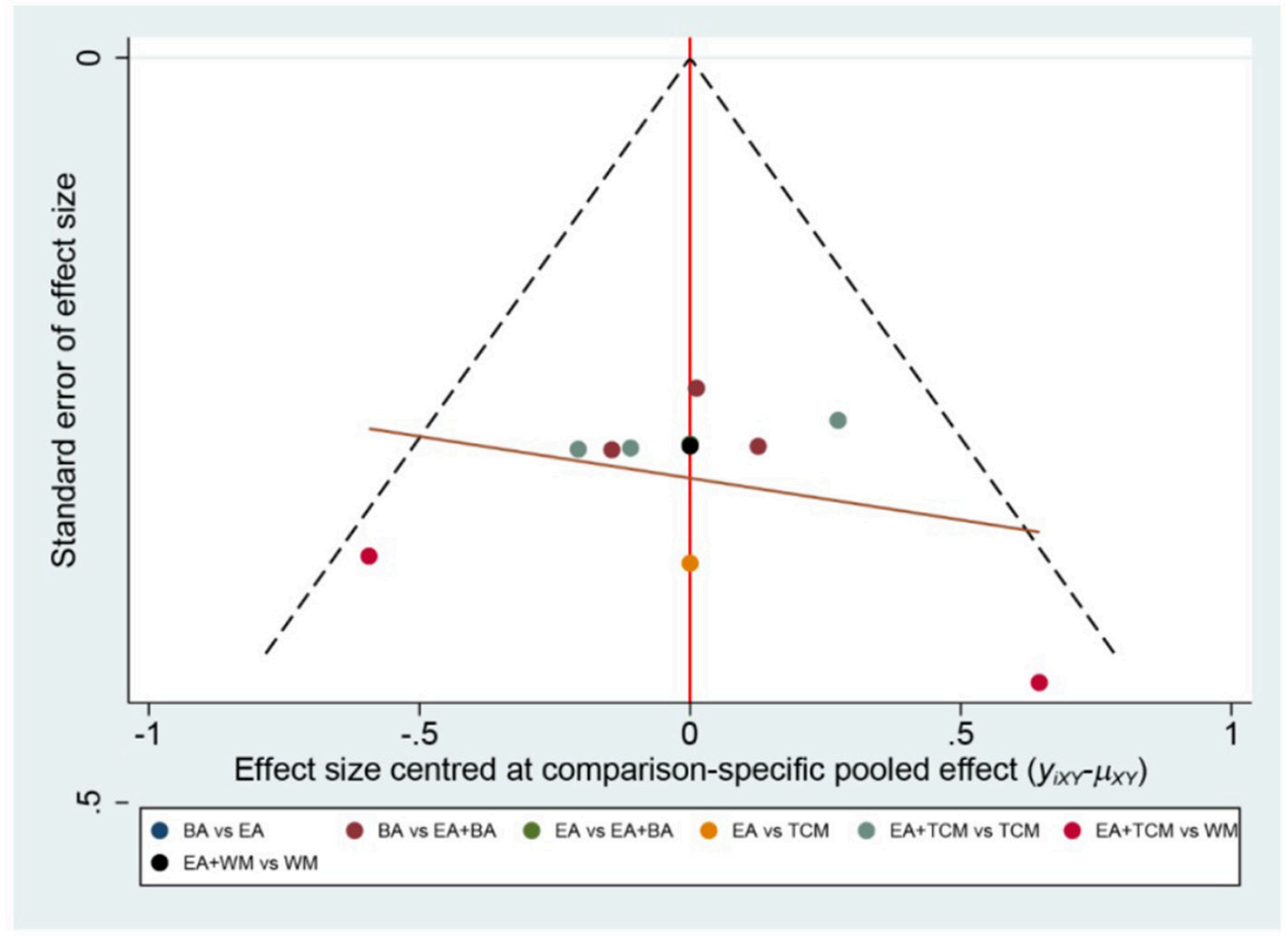
Comparative-corrected funnel plot of CMSCS.

The SUCRA probability ranking indicates the following sequence: EA+BA (97.4%) > EA (83.1%) > BA (69.5%) > EA+TCM (50.0%) > TCM (33.3%) > EA+WM (15.2%) > WM (1.5%). These results suggest that the combination of eye acupuncture with body acupuncture may provide the most effective intervention for reducing CMSCS. The SUCRA probability ranking indicates the following sequence: EA+BA (97.4%) > EA (83.1%) > BA (69.5%) > EA+TCM (50.0%) > TCM (33.3%) > EA+WM (15.2%) > WM (1.5%). These results recommend that the combination of eye acupuncture with body acupuncture may supply the most effective intervention for reducing CMSCS. Figure 13 shows the SUCRA possibility position. Furthermore, the “comparative-corrected” funnel story recommends the visibility of some small sample result and publication bias, as depicted in Figure 14.

### 3.5 Adverse reactions

Among the 31 studies, the majority (*n* = 23) did not actively monitor or mention adverse events. Four studies (21, 27, 29, 36) explicitly stated that no adverse events occurred during their trial periods. A total of 4 studies (23, 37, 39, 45) documented minor adverse reactions. The incidence of these events was below 20% in all reported cases, and no statistically significant difference was observed between the intervention and control groups (*P* > 0.05). None of the studies reported participant withdrawals due to Adverse Reactions. Detailed information on Adverse Reaction monitoring, definitions, severity, and withdrawals for each study is summarized in Supplementary Table 2.

## 4 Discussion

Insomnia represents a common sleep disorder frequently encountered in clinical practice, and it significantly diminishes individuals' quality of life and is highly prevalent in the general population. EA therapy, pioneered by Professor Jing-Shan Peng, utilizes micro-needle therapy to target acupoints around the eyes for disease treatment. Renowned for its precise acupoint targeting and ease of application, EA has been practiced at Liaoning University of Traditional Chinese Medicine for over four decades, benefiting numerous insomnia patients and demonstrating significant clinical significance. Today, EA finds broad application across various clinical domains, including cerebrovascular disease, pain management, neurological disorders, and mental health conditions (52). However, despite its widespread use, the clinical application of EA remains complex, with insufficient direct or indirect evidence to comprehensively assess its effects on insomnia intervention. Therefore, adhering to the PRISMA statement, and employing a systematic review and network meta-analysis approach, this study aimed to assess the efficacy and safety of eye acupuncture in comparison to alternative treatments for managing insomnia. Multiple outcome indicators were utilized to comprehensively evaluate the effectiveness of EA in insomnia intervention.

In this study, we systematically examined 31 randomized controlled trials involving 9 interventions targeting insomnia. Our analysis aimed to evaluate the effectiveness of these interventions in addressing insomnia symptoms, as measured by outcomes including Clinical Effectiveness, PSQI score, and CMSCS. Using network meta-analysis, we comprehensively evaluated the efficacy and safety of various EA therapies, both as monotherapy and in combination with other treatment modalities, for the management of insomnia. The findings indicate that interventions involving EA consistently demonstrated superior outcomes in terms of Clinical Effectiveness, PSQI scores, and CMSCS compared to non-EA interventions. Notably, the combination therapies, particularly EA+BA and EA+WA, emerged as the most promising approaches, ranking highest in SUCRA values for PSQI/CMSCS and Clinical Effectiveness, respectively.

The observed differential ranking patterns between EA+BA and EA+WA across outcomes may be attributed to distinct mechanistic emphases. EA+BA potentially offers a synergistic effect by modulating both central and systemic pathways. EA may primarily influence cerebral blood flow and neurovascular coupling (16, 17). while BA is known to regulate limbic system activity and HPA axis function via the spinothalamic-cortical pathway (18). This combined central and systemic regulation might lead to broader improvements in sleep architecture and somatic symptoms, reflected in the superior performance of EA+BA on the PSQI and CMSCS. In contrast, WA is a modern micro-acupuncture technique renowned for its potent analgesic and sedative effects, potentially mediated by inhibiting sympathetic nervous system activity and modulating spinal cord and brainstem signal processing (53). The combination of EA's central regulation with WA's strong sedative properties might create a powerful, rapid-acting neuro-sedative effect, making it exceptionally effective according to the criteria of Clinical Effectiveness, which often prioritizes immediate and tangible symptomatic relief. Furthermore, the finding that EA alone sometimes performed comparably to combination therapies in certain comparisons might be attributed to the fundamental role of periocular stimulation in modulating key neurovascular and potentially neurotransmitter systems involved in sleep initiation and maintenance.

Several important limitations of this study must be considered when interpreting the results. First, the majority of the included trials presented a high risk of bias, primarily due to inadequate reporting of randomization procedures and a widespread lack of allocation concealment and blinding. These methodological shortcomings likely lead to an overestimation of treatment effects, particularly for subjective patient-reported outcomes like PSQI and CMSCS, which are highly susceptible to performance and detection bias. We initially planned a sensitivity analysis excluding high-risk studies; however, this was not feasible due to the uniformly low quality of the majority of included studies and the limited number of eligible alternatives. Consequently, the high risk of bias across studies substantially limits the certainty of the treatment rankings derived from SUCRA. The reported SUCRA values and their differences (e.g., separations of 5%−10%) should be interpreted with caution, as they may not reliably reflect clinically meaningful distinctions given the underlying methodological limitations. Second, the asymmetry observed in the funnel plots for primary outcomes (e.g., PSQI) suggests the potential presence of publication bias and small-study effects. This may have inflated the effect estimates and SUCRA rankings for certain interventions, particularly those comparisons reliant on a smaller number of trials. Future updates incorporating more high-quality, large-sample trials are needed to mitigate these biases. Third, the definition of “Clinical Effectiveness” based on percentage reduction in PSQI, while commonly used in Chinese RCTs, lacks universal standardization and validation against established minimal clinically important difference (MCID) thresholds. As summarized in Supplementary Table 1, variations in the specific criteria for “cure” and “effective” across studies introduce heterogeneity and threaten comparability. Moreover, percentage change is inherently dependent on baseline scores, which were not adjusted for in our analysis, potentially influencing results, especially in studies with severely impaired populations. Fourth, significant clinical heterogeneity may exist due to variations in acupuncture techniques, treatment durations, and practitioner experience, which were not fully accounted for. Fifth, the body of evidence is comprised exclusively of small-scale, methodologically limited RCTs from China, which substantially limits the generalizability of our findings. Sixth, Eye acupuncture lacks a standardized protocol, and its efficacy is influenced by practitioner experience, which precluded quantitative assessment. Finally, safety data were insufficiently reported, with only a few trials documenting adverse events using inconsistent definitions and monitoring methods. The high probability of under-reporting bias weakens the conclusion that EA is “safe.” Future studies should adopt standardized safety reporting frameworks (e.g., STRICTA, CONSORT-Harms) to improve transparency.

## 5 Conclusions

Based on the aforementioned findings, the current evidence suggests that EA may have clinical value in treating insomnia. The analysis indicates that its application, compared to non-utilization, is associated with superior therapeutic efficacy in ameliorating insomnia symptoms among affected individuals in the included studies. Moreover, EA+BA showed the highest probability of being the optimal intervention for improving sleep quality and reducing Chinese medicine symptom clusters, while EA+WA ranked first in terms of clinical effectiveness. These integrated approaches not only enhance clinical efficacy but also effectively alleviate insomnia symptoms. Furthermore, being non-pharmacological treatment modalities, combination therapies circumvent the adverse effects associated with drug usage, thus offering safer alternatives for patients. However, the results should be interpreted with caution due to the methodological limitations of the included studies, including suboptimal randomization procedures and lack of blinding. Future rigorously designed, large-scale, and multicenter randomized controlled trials incorporating objective sleep measurements and long-term follow-up are warranted to validate these findings and further elucidate the underlying mechanisms.

## Data Availability

The original contributions presented in the study are included in the article/Supplementary material, further inquiries can be directed to the corresponding authors.
