# Supplementary material for: Eye acupuncture therapy for insomnia: a systematic review and network meta-analysis
Source: Front Neurol. 2025 Dec 5;16:1720073. doi: 10.3389/fneur.2025.1720073 (PMC12714621; doi:10.3389/fneur.2025.1720073)
Supplement: Supplementary file 1 [file Table_1.docx]

**Table S1. Summary of “effectiveness” definitions in included studies**

| **Study ID** | **Criteria for “Cure” (PSQI-based)** | **Criteria for “Effective” (PSQI-based)** | **Criteria for “Ineffective” (PSQI-based)** |
| --- | --- | --- | --- |
| Zhao 2022 | PSQI score reduction rated ≥ 75% | PSQI score reduction rated ≥ 25% | PSQI score reduction rate ＜25% |
| Bai and Hai 2021 | PSQI score reduction rated ≥ 70% | PSQI score reduction rated ≥ 30% | PSQI score reduction rate ＜30% |
| Bai 2021 | PSQI score reduction rated ≥ 70% | PSQI score reduction rated ≥ 30% | PSQI score reduction rate ＜30% |
| Luo 2020 | PSQI score reduction rated ≥ 75% | PSQI score reduction rated ≥ 25% | PSQI score reduction rate ＜25% |
| Qin 2019 | PSQI score reduction rated ≥ 70% | PSQI score reduction rated ≥ 30% | PSQI score reduction rate ＜30% |
| Li 2019 | PSQI score reduction rated ≥ 75% | PSQI score reduction rated ≥ 25% | PSQI score reduction rate ＜25% |
| Li and Wang 2019 | PSQI score reduction rated ≥ 75% | PSQI score reduction rated ≥ 25% | PSQI score reduction rate ＜25% |
| Huang 2019 | PSQI score reduction rated ≥ 75% | PSQI score reduction rated ≥ 25% | PSQI score reduction rate ＜25% |
| Zhang 2018 | PSQI score reduction rated ≥ 75% | PSQI score reduction rated ≥ 25% | PSQI score reduction rate ＜25% |
| Cao 2020 | PSQI score reduction rated ≥ 70% | PSQI score reduction rated ≥ 30% | PSQI score reduction rate ＜30% |
| Wang and Wang 2018 | PSQI score reduction rated ≥ 75% | PSQI score reduction rated ≥ 25% | PSQI score reduction rate ＜25% |
| Wang 2018 | PSQI score reduction rated ≥ 75% | PSQI score reduction rated ≥ 25% | PSQI score reduction rate ＜25% |
| Hu 2018 | PSQI score reduction rated ≥ 75% | PSQI score reduction rated ≥ 25% | PSQI score reduction rate ＜25% |
| Liu 2017 | PSQI score reduction rated ≥ 75% | PSQI score reduction rated ≥ 25% | PSQI score reduction rate ＜25% |
| Ma 2016 | PSQI score reduction rated ≥ 70% | PSQI score reduction rated ≥ 30% | PSQI score reduction rate ＜30% |
| Tian 2015 | PSQI score reduction rated ≥ 75% | PSQI score reduction rated ≥ 25% | PSQI score reduction rate ＜25% |
| Cheng 2015 | PSQI score reduction rated ≥ 70% | PSQI score reduction rated ≥ 30% | PSQI score reduction rate ＜30% |
| Xu 2014 | PSQI score reduction rated ≥ 75% | PSQI score reduction rated ≥ 25% | PSQI score reduction rate ＜25% |
| Zhang 2013 | PSQI score reduction rated ≥ 95% | PSQI score reduction rated ≥ 30% | PSQI score reduction rate ＜30% |
| Liu 2013 | PSQI score reduction rated ≥ 75% | PSQI score reduction rated ≥ 25% | PSQI score reduction rate ＜25% |
| Cui 2011 | PSQI score reduction rated ≥ 75% | PSQI score reduction rated ≥ 25% | PSQI score reduction rate ＜25% |
| Luo 2010 | PSQI score reduction rated ≥ 95% | PSQI score reduction rated ≥ 30% | PSQI score reduction rate ＜30% |
| Huang 2010 | PSQI score reduction rated ≥ 70% | PSQI score reduction rated ≥ 30% | PSQI score reduction rate ＜30% |

**Table S2. Summary of adverse event (AE) reporting across included studies**

| **Study** | **AE Monitored** | **AE Definitions** | **AE Severity** | **Withdrawals Due to AEs** |
| --- | --- | --- | --- | --- |
| Zhang 2021 | Yes | Fainting from needles, pain, needle retention, hematoma | Mild | No |
| Wang 2018 | Yes | Taste disorders, dizziness | Mild | No |
| Guo 2018 | Yes | Subcutaneous bruise | Mild | No |
| Xu 2014 | Yes | Dizzy | Mild | No |
| Zhao 2022 | Yes | No AEs observed | - | - |
| Cao 2020 | Yes | No AEs observed | - | - |
| Qin and Chen 2019 | Yes | No AEs observed | - | - |
| Wang and Wang 2018 | Yes | No AEs observed | - | - |
| Li 2022 | No | - | - | - |
| Bai and Hai 2021 | No | - | - | - |
| Bai 2021 | No | - | - | - |
| Luo and Yang 2020 | No | - | - | - |
| Wu 2019 | No | - | - | - |
| Li Qin 2019 | No | - | - | - |
| Li and Wang 2019 | No | - | - | - |
| Li 2019 | No | - | - | - |
| Huang 2019 | No | - | - | - |
| Zhang 2018 | No | - | - | - |
| Xie 2018 | No | - | - | - |
| Hu 2018 | No | - | - | - |
| Wang 2017 | No | - | - | - |
| Li 2017 | No | - | - | - |
| Ma 2016 | No | - | - | - |
| Tian 2015 | No | - | - | - |
| Cheng 2015 | No | - | - | - |
| Cheng 2014 | No | - | - | - |
| Zhang 2013 | No | - | - | - |
| Liu 2013 | No | - | - | - |
| Cui 2011 | No | - | - | - |
| Luo 2010 | No | - | - | - |
| Huang 2010 | No | - | - | - |
